# Colour Patterns Do Not Diagnose Species: Quantitative Evaluation of a DNA Barcoded Cryptic Bumblebee Complex

**DOI:** 10.1371/journal.pone.0029251

**Published:** 2012-01-06

**Authors:** James C. Carolan, Tomás E. Murray, Úna Fitzpatrick, John Crossley, Hans Schmidt, Björn Cederberg, Luke McNally, Robert J. Paxton, Paul H. Williams, Mark J. F. Brown

**Affiliations:** 1 Department of Biology, National University of Ireland Maynooth, Maynooth, Ireland; 2 Department of Zoology, School of Natural Sciences, Trinity College Dublin, Dublin, Ireland; 3 Institute for Biology, Martin-Luther-Universität Halle-Wittenberg, Halle, Germany; 4 School of Biological Sciences, Queen's University Belfast, Belfast, United Kingdom; 5 National Biodiversity Data Centre, Carriganore, WIT West Campus, Waterford, Ireland; 6 North Flaws, South Ronaldsay, Orkney, United Kingdom; 7 Tjørnevej, Holstebro, Denmark; 8 Swedish Species Information Centre, University of Agricultural Sciences, Uppsala, Sweden; 9 Department of Entomology, The Natural History Museum, London, United Kingdom; 10 School of Biological Sciences, Royal Holloway University of London, London, United Kingdom; Biodiversity Insitute of Ontario - University of Guelph, Canada

## Abstract

Cryptic diversity within bumblebees (*Bombus*) has the potential to undermine crucial conservation efforts designed to reverse the observed decline in many bumblebee species worldwide. Central to such efforts is the ability to correctly recognise and diagnose species. The *B. lucorum* complex (*Bombus lucorum*, *B. cryptarum* and *B. magnus*) comprises one of the most abundant and important group of wild plant and crop pollinators in northern Europe. Although the workers of these species are notoriously difficult to diagnose morphologically, it has been claimed that queens are readily diagnosable from morphological characters. Here we assess the value of colour-pattern characters in species identification of DNA-barcoded queens from the *B. lucorum* complex. Three distinct molecular operational taxonomic units were identified each representing one species. However, no uniquely diagnostic colour-pattern character state was found for any of these three molecular units and most colour-pattern characters showed continuous variation among the units. All characters previously deemed to be unique and diagnostic for one species were displayed by specimens molecularly identified as a different species. These results presented here raise questions on the reliability of species determinations in previous studies and highlights the benefits of implementing DNA barcoding prior to ecological, taxonomic and conservation studies of these important key pollinators.

## Introduction

Pollination is a key ecosystem service for both natural and agricultural ecosystems. While evidence for a pollination crisis is equivocal [1], [2], [3], it is increasingly clear that wild pollinators, and particularly bees, are in decline worldwide [4], [5], [6], [7]. Consequently, a key aim in insect conservation is to understand and reverse these declines. In northern temperate regions, bumblebees – as ecologically and commercially important pollinators – have been the focus of considerable conservation effort [5], [8], [9], [10], [11], [12]. Conservation of any group requires an assessment of both current status and the impact of potential interventions, and this in turn requires accurate species identification.

While recent phylogenetic analyses have advanced our understanding of bumblebee taxonomy [13], *Bombus* species are notorious for possessing convergent colour patterns, resulting in cryptic species [14], [15]. Morphologically cryptic species pose significant problems for field assessment of species richness and diversity, both of which are central to understanding current and future population trends of these important pollinators. Unfortunately, in Western Europe one of the apparently most common bumblebees, *B. lucorum* (Linnaeus), belongs to such a cryptic species complex. While *B. lucorum s.l.* is common throughout Europe [16], [17], [18], and has been commercially reared as a pollinator for greenhouse crops [19], increasing chemical [20] and molecular [20], [21], [22], [23] evidence supports the idea that it actually represents three distinct species, *B. cryptarum* (Fabricius), *B. lucorum* and *B. magnus* (Vogt). The use of molecular tools in large-scale ecological studies has revealed differences in the ecology, abundance and distribution of these putative species [22], [23], indicating the need to reassess the conservation status and response of these species to interventions separately.

Presently, the majority of species records are collected by members of the public using morphological and especially colour-pattern characters for identification (e.g., in the UK by the Bees, Wasps and Ants Recording Scheme, www.bwars.com and the Bumblebee Conservation Trust http://www.bumblebeeconservation.org.uk/). Therefore, there is a clear need to assess the value of colour-pattern characters in distinguishing these species. Both the scientific and popular literature suggest that such characters can be used to identify colony-founding queens in the spring [20], [24], [25], [26], [27], [28], [29]. Morphology based diagnosis of *B. lucorum s.l.* queens principally involves characteristics of the collar, labrum, ocelli and metasomal terga [20], [24], [30], [31]. Of these, the most commonly used characters for species diagnosis are those describing the pile (‘hair’) of the collar (a band of yellow hairs at the anterior of the thorax) [24], [25], [26], [27], particularly: (i) the extent of a collar extension below the tegula [24]; (ii) the presence or absence of melanisation in the collar [20]; and (iii) the presence or absence of an ‘S-shape’ of black hairs on the side of the collar [20]. While the extension of such traits to identifying workers has been shown to fail [23], and despite reservations that character variation may not be discrete [28], to our knowledge there has been no quantitative assessment of their accuracy in identifying queens. The main aim of this study is to use molecular tools to assess the value of these popular colour-pattern characters in biodiversity and conservation surveys of the *B. lucorum* species complex.

The DNA barcode, an approximately 650 bp region of the cytochrome c oxidase (COI) mitochondrial gene, has become an invaluable tool for species identification and taxonomic hypothesis generation for both ecologists and taxonomists alike [32], [33], [34], [35]. To date over 700,000 barcode records have been generated for insects (http://www.boldsystems.org) and barcoding has been utilised across major insect orders including Lepidoptera [36], [37], [38], [39], Coleoptera [40] Diptera [34], [41], [42], [43], Hemiptera [44], [45], [46] and Hymenoptera [47], [48], [49]. In many cases the morphological characters traditionally used to describe a species or indicate relationships among species have been re-evaluated in the light of groupings arising from DNA barcoding studies. Evaluating species diagnostic morphological characters of genetically discrete units in such a way can indicate strengths and weaknesses in current taxonomic diagnoses of the group under study and often taxonomic revision may follow [35], [44], [49], [50].

However, for the *lucorum* complex the diagnostic value of these characters has yet to be assessed quantitatively against independently determined specimens. Here we combine DNA barcoding and quantitative colour-pattern analyses to address this question at the scale of both Ireland and continental Europe.

## Materials and Methods

### Taxon sampling and morphological analysis

Irish specimens used in this study were obtained from a collection originally sampled in 2005 and 2006 [22]. In order to extend the study to a European scale, and investigate geographic variance, specimens were obtained from the Orkney Islands of Northern Scotland, Denmark and Finland. Field studies did not involve endangered or protected species and no specific permits were required for the collection of specimens included in this study. Permission was not required for the collection of Danish, Scottish, Finnish and the majority of Irish specimens. Collecting unlisted insect species samples from lands of the Irish National Parks and Botanic Gardens required only verbal permission, and this was obtained from the National Park Rangers for each park, and from Dr Peter Wyse Jackson, Director of the National Botanic Gardens. Queens of *B. lucorum* agg. were collected in all regions, except Denmark, without prior morphological identification. Danish queens were initially morphologically identified to characterise representative specimens of each putative species prior to our genetic analyses. As such, specimens that displayed characters at the extremes of the morphological ranges were most likely to occur in the Danish samples. In total 67 queens were included (Table S1) in the molecular and morphometric analyses. The Irish and Danish groups (totaling 48 queens) were also analysed separately, as they consisted of all three species (see below). Using electronic Vernier calipers (Mitutoyo Absolute Digimatic), we measured thorax width between the tegulae (body size), breadth of the collar on top of the thorax, vertical and horizontal extensions of the collar below the tegula, and breadth of the collar below the tegula. We also recorded a series of qualitative characters that have been deemed to be diagnostic for the three species: (i) the presence or absence of a black ‘S-shape’ on the side of the collar; (ii) the presence or absence of melanisation in the collar; and (iii) the presence of a collar extension below the tegula. To account for variation in quantitative morphological characters that may be affected by animal size, relative sizes were calculated using thorax breadth as a measure of body size. Morphological data were analysed using either one-way ANOVAs with Tukey's post-hoc test for pairwise differences, or G-tests. All analyses were conducted using SPSS v. 16.0. Data are shown as boxplots as we are interested in the biological variation in morphological traits.

### DNA extraction and amplification

Total DNA was extracted from the mid or hind legs using the DNeasy Blood & Tissue extraction kit (Qiagen), with a modified protocol. Legs were ground in a 1.5 ml Eppedorf tube comprising 200 µl PBS with a disposable polypropylene pestle connected to a hand held motor (Sigma Aldrich). Then 180 µl of Buffer AL and 20 µl Proteinase K (Qiagen kit components) were added and the sample was homogenized further prior to incubation at 70°C for 30 mins. Thereafter, 200 µl of 100% ethanol was added and the remaining extraction followed the manufacturer's protocol.

For amplification and sequencing of the COI region the primers LCOHym (5′–TATCAACCAATCATAAAGATATTGG–3′; [51]) and NancyShort were used (5′ CCCGGTAAAATTAAAATATAAAC-3′; [52]. PCRs were carried out in 20 µl volumes using 1× PCR buffer (Invitrogen), 2.5 mM MgCl_2_, 0.2 mM of each primer, 0.2 mM of each dNTP, 1 U Taq polymerase (Invitrogen) and approximately 50 ng template DNA. PCR reaction conditions included denaturation at 94°C for 240 s followed by 32 cycles of 60 s at 94°C, 45 s at 48°C, 60 s at 72°C and a final extension at 72°C for 420 s in a Peltier thermal cycler (PTC 200; MJ Research). PCR products were sequenced in the School of Natural Sciences, Trinity College using an ABI 3130xl capillary automated sequencers (Applied Biosystems Inc.). The same PCR primers were used for sequencing. Forward and reverse sequence reads were view and assembled into contiguous sequences using BioEdit v7.0.9 [53]. All polymorphic sites were visually inspected to ensure correct base calling and sequences were aligned manually. The electropherogram, sequence and specimen data were submitted to BOLD (Accessions JCLUC001-11 to JCLUC-067-11 http://www.barcodinglife.org) and are available in the “Irish *Bombus lucorum*” project folder of BOLD. Sequences were also deposited in GenBank (Accessions JN872566 to JN872632).

### Sequence Analysis

The final aligned matrix was imported into MEGA version 4.0 [54] for phylogenetic analysis. No gaps were present and translations of the sequences indicated the absence of stop and nonsense codons. After sequence trimming a total of 642 base pairs were available for analysis. Nucleotide positions were determined using the full COI gene sequence from the mitochondrial genome of *B. hypocrita* (GenBank accession no. NC011923; positions 1996 to 3555) and the mouse COI reference sequence (derived from GenBank accession no. AK166798). Sequence divergences were calculated using the Kimura 2-parameter (K2P) distance model [55] using MEGA 4. Bayesian analysis was set and implemented using Bayesian Evolutionary Analysis Utility (BEAUti) v 1.5.4 and Bayesian Evolutionary Analysis Sampling Trees (BEAST; [56] v 1.5.4 using the general time reversible and gamma (GTR+G) substitution model, uncorrelated lognormal relaxed clock (UCLN) model and coalescent tree prior. *B. terrestris* (Linnaeus; BOLD accession 6876C01; Williams PH, An J, Brown MJF, Carolan JC, Goulson D, Huang J and Ito M, An unsuspected cryptic bumblebee: consequences for conservation and the trade in greenhouse pollinators, submitted) was chosen to root the trees based on the results of Cameron et al., [13] and Murray et al., [22] which indicate its sister group status with respect to the taxa included in this study. The analysis was run for 10,000,000 generations with sampling of trees every 1000 generations. After termination the Markov chain Monte Carlo (MCMC) output was analysed using TreeAnnotator v.1.5.4 (http://beast.bio.ed.ac.uk) to produce a consensus tree from the post burn-in tree sample (burn-in 1000) with a posterior probability of 0.5, targeting the maximum clade credibility tree and keeping the target node heights. All trees were opened and viewed in FigTree v 1.3 (http://tree.bio.ed.ac.uk/software/figtree/). Haplotype variation, diagnostic sequence distribution and inter- and intra-specific divergences were determined using the HapMap application of the iBarcode website (http://www.iBarcode.org/).

## Results

**Figure 1:**
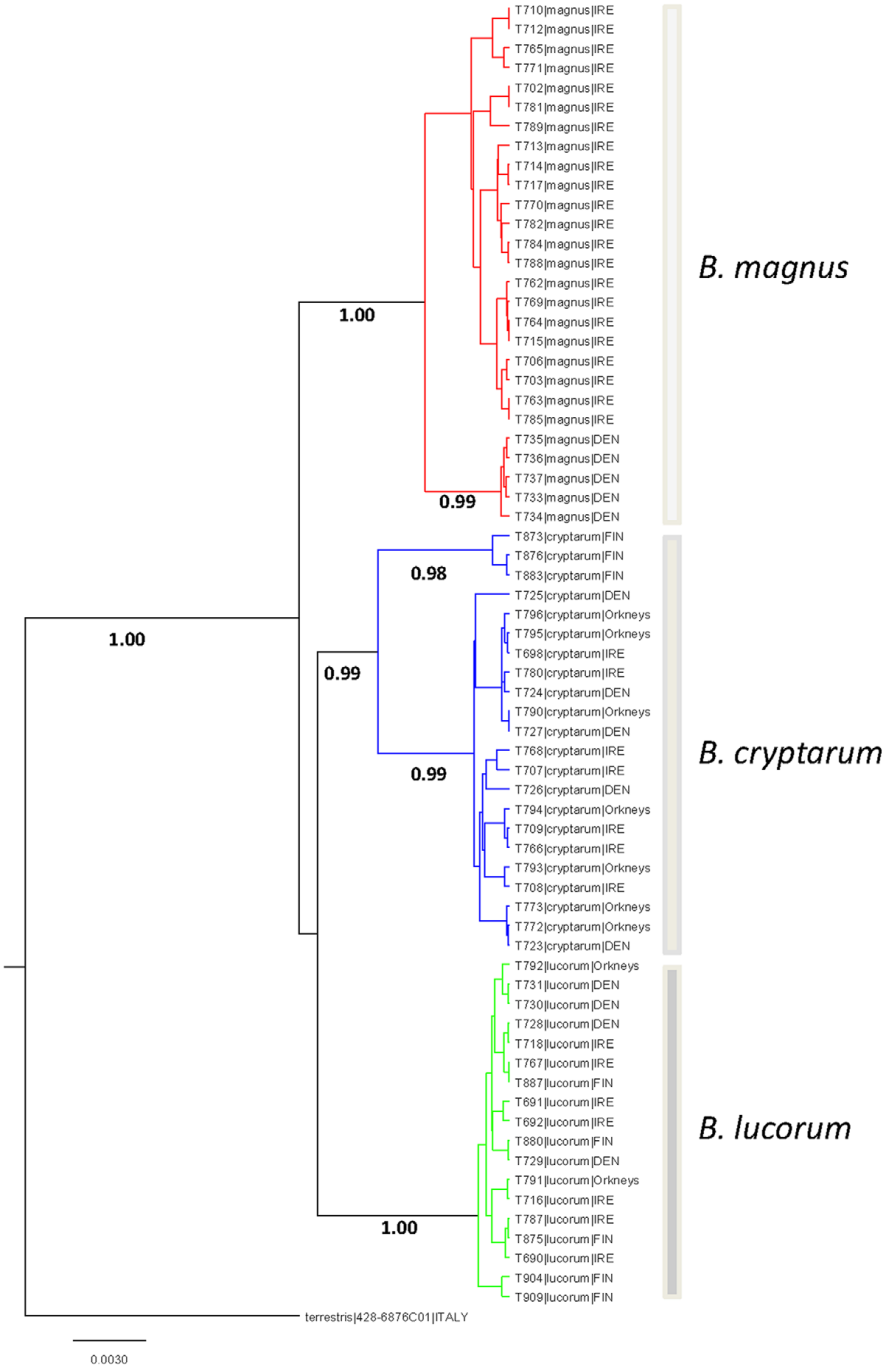
Consensus Bayesian tree generated from 642 bps of the mitochondrial COI region for 67 representatives of the *lucorum* complex. Bayesian MCMC analysis was conducted using the general time reversible model, gamma distribution and 1 million generations. Numbers below the branches indicate posterior probabilities. Scale bar relates to expected number of substitutions per site. *B. terrestris* (428–6876COI) was included as an out-group and used to root the trees.

The COI barcode was sequenced for 67 representatives of the *lucorum* complex with the majority of the barcode sequence being obtained for each. The reference DNA barcode begins 58 bp from the 5′- end of the mouse COI gene, which corresponds to position 52 of the *B. hypocrita* COI gene. In total, 642 nucleotides were available for analysis. Bayesian analyses resulted in a tree (Figure 1) with three distinct and well supported clades, each of which we assume represents one of the three species. Of the 37 Irish specimens, 22 were *B. magnus,* 8 were *B. cryptarum,* and 7 were *B. lucorum*. The Danish *B. magnus* resolved as a sister group to Irish *B. magnus* (Clade 1; 1.00 Bayesian Posterior Probability, (BPP)), differentiated by two polymorphisms that represent synonymous substitutions. Danish, Irish and Orkney Island *B. cryptarum* resolved as a monophyletic group with Finnish representatives, resolving as a sister group to the main group (Clade 2; 0.99 BPP). Finnish specimens possessed 8 polymorphisms with respect to their Irish/Orkney/Danish counterparts. Only a single polymorphism (C/T at position 391) was evident within the *B. lucorum* clade (Clade 3; 1.00 BPP) and this polymorphism was shared by two Finnish specimens (T904 and T909).

In total, there were 50 variable characters of which 41 were deemed to be informative. Haplotype mapping using iBarcode web resources indicated that 16 haplotypes were present across all species. However, a number of these are derived from unique nucleotide polymorphisms found in a single specimen. Such haplotypes were discounted from further consideration and specimens with a single polymorphism were included in the counts for the most similar haplotype. Two distinct haplotypes are evident for *B. magnus* and *B. cryptarum,* where the haplotypes correspond to geographically distinct groups. Of the seven nucleotide differences between the two *B. cryptarum* haplotypes, four are shared between the Finnish *B. cryptarum* and both *B. magnus* and *B. lucorum*. Although a number of diagnostic nucleotide characters were evident for each species, when each was compared to the other two species only two nucleotide positions can be deemed diagnostic for species delineation across the entire complex (Table 1). They are at position 203 (Thymine, Cytosine and Adenine for *B. cryptarum, B. lucorum* and *B. magnus* respectively) and position 553 (Adenine, Thymine and Cytosine for *B. cryptarum, B. lucorum* and *B. magnus* respectively). Within species, genetic distances for *B. cryptarum, B. lucorum* and *B. magnus* were 0.004, 0.001 and 0.001 respectively. Interspecific genetic distances were considerably higher and ranged from 0.033 to 0.044.

**Table 1:** COI haplotypes, polymorphic sites and codon positions for *B. cryptarum, B. lucorum* and *B. magnus*.

	Polymorphic Site																				
Haplotype	1	34	40	50	65	79	97	98	124	139	169	193	203	205	211	226	262	278	280	284	293
*B. cryptarum* haplotype 1 (19)	T	T	T	T	T	A	T	C	A	A	T	T	**T**	A	T	C	T	C	T	C	A
*B. cryptarum* haplotype 2 (3)		C										C				T					
*B. lucorum* haplotype 1 (16)			A	C					T		C		**C**		A			T	A	T	T
*B. lucorum* haplotype 2 (2)			A	C					T		C		**C**		A			T	A	T	T
*B. magnus* haplotype 1 (22)	C				C		C						**A**	T	A	T	A	T	A	T	
*B. magnus* haplotype 2 (5)	C				C	G	C			G			**A**	T	A	T	A	T	A	T	

*continued*	Polymorphic Site																				
Haplotype	298	313	328	331	346	358	364	391	403	475	490	496	538	553	565	595	607	616	619	625	637
*B. cryptarum* haplotype 1 (19)	T	A	A	T	T	C	A	T	A	T	T	T	C	**A**	A	T	T	A	G	T	T
*B. cryptarum* haplotype 2 (3)	C					T							T				C		A	C	
*B. lucorum* haplotype 1 (16)		T	T	C	A	T	C	C			C		T	**T**	T	C	C	T	A		
*B. lucorum* haplotype 2 (2)		T	T	C	A	T	C				C		T	**T**	T	C	C	T	A		
*B. magnus* haplotype 1 (22)						T			T	C		C	T	**C**			C		A		C
*B. magnus* haplotype 2 (5)						T			T	C		C	T	**C**			C		A		C

Number of individuals in parenthesis. Nucleotides in bold represent fixed diagnostic polymorphisms for each species.

### Morphological analysis

Quantitative measurements and qualitative scores were obtained for a range of morphological characters that have been suggested to be diagnostic for species or of taxonomic importance for *B. lucorum s.l.*.

### Body size

**Figure 2:**
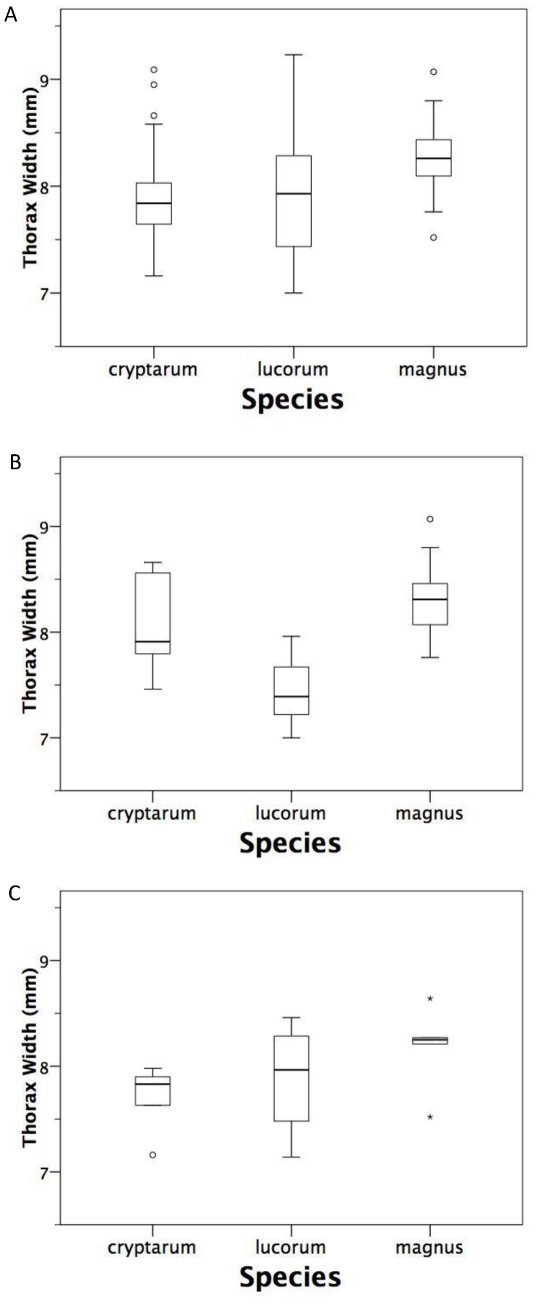
Body size, measured as thorax width, in *B. cryptarum*, *B. lucorum* and *B. magnus* queens (a) for all samples combined, (b) for Irish specimens, and (c) for Danish specimens. Boxplots show the median, upper and lower quartiles, 99% confidence limits and outliers.

While there was considerable overlap among species in body size (Fig. 2a), there were nevertheless significant differences among the species in mean thorax breadth between the tegulae (Tables 2, 3). Post-hoc tests showed that *B. magnus* was bigger than *B. lucorum* (Table 2, *P* = 0.035) and *B. cryptarum* (Table 2, *P* = 0.043), but there was no difference between the latter two species (*P* = 0.943; Fig. 2a, Table 2). Analysing the Irish specimens on their own, there was still a significant difference in body size (Tables 2, 3), with post-hoc tests showing that *B. magnus* was bigger than *B. lucorum* (Table 2, *P*<0.001) but not *B. cryptarum* (Table 2, *P* = 0.438), and that *B. cryptarum* was bigger than *B. lucorum* (Table 2, *P* = 0.014; Fig. 2b). Finally, analysing the Danish specimens on their own, there was no significant difference in body size across all species or in any of the pairwise comparisons (Tables 2, 3, all *P*>0.2; Figure 2c).

**Table 2:** Morphological measurements for molecularly identified queens of *Bombus cryptarum*, *B. lucorum* and *B. magnus* (mean±SD).

	All samples			Ireland samples			Denmark samples		
	*cryptarum*	*lucorum*	*magnus*	*cryptarum*	*lucorum*	*magnus*	*cryptarum*	*lucorum*	*magnus*
Thorax width (mm)	7.96±0.50	7.91±0.60	8.28±0.35	8.11±0.48	7.45±0.38	8.14±0.34	7.70±0.33	7.88±0.56	8.18±0.41
Collar breadth – top (mm) A	2.80±0.45	2.79±0.42	2.87±0.31	2.94±0.22	2.51±0.46	2.87±0.31	2.43±0.56	2.62±0.27	2.89±0.33
Relative A	0.35±0.06	0.35±0.05	0.35±0.42	0.36±0.04	0.34±0.07	0.35±0.04	0.31±0.07	0.33±0.05	0.33±0.06
Length of collar below tegula – right (mm) B	1.43±0.46	0.78±0.41	2.55±0.56	1.61±0.32	0.46±0.42	2.61±0.56	1.33±0.77	0.86±0.23	2.25±0.49
Length of collar below tegula – left (mm) C	1.67±0.41	0.93±0.49	2.58±0.42	1.81±0.24	0.47±0.44	2.60±0.42	2.01±0.43	1.02±0.09	2.45±0.41
Length of collar below tegula – average (mm) D	1.55±0.38	0.85±0.44	2.56±0.42	1.71±0.27	0.47±0.43	2.61±0.41	1.67±0.54	0.94±0.16	2.36±0.41
Relative B	0.18±0.06	0.10±0.05	0.31±0.07	0.20±0.05	0.06±0.06	0.32±0.07	0.17±0.10	0.11±0.03	0.28±0.07
Relative C	0.21±0.05	0.12±0.06	0.31±0.05	0.23±0.04	0.07±0.06	0.31±0.05	0.26±0.05	0.13±0.01	0.30±0.05
Relative D	0.20±0.05	0.11±0.05	0.31±0.06	0.21±0.04	0.06±0.06	0.31±0.06	0.22±0.07	0.12±0.02	0.29±0.06
Breadth of collar below tegula – right (mm) E	2.11±0.55	1.66±0.74	2.93±0.38	2.29±0.24	1.31±1.20	2.95±0.31	1.78±1.00	1.48±0.08	2.87±0.63
Breadth of collar below tegula – left (mm) F	2.21±0.34	1.66±0.79	2.81±0.32	2.15±0.20	1.27±1.22	2.84±0.34	2.47±0.13	1.47±0.29	2.67±0.21
Breadth of collar below tegula – average (mm) G	2.16±0.33	1.66±0.75	2.87±0.31	2.22±0.18	1.29±1.20	2.89±0.29	2.12±0.52	1.47±0.17	2.77±0.42
Relative E	0.27±0.07	0.21±0.10	0.36±0.05	0.28±0.04	0.18±0.17	0.36±0.04	0.23±0.13	0.19±0.00	0.35±0.08
Relative F	0.28±0.05	0.21±0.10	0.34±0.04	0.27±0.04	0.18±0.17	0.34±0.04	0.32±0.03	0.19±0.03	0.33±0.03
Relative G	0.27±0.05	0.21±0.10	0.35±0.04	0.28±0.04	0.18±0.17	0.35±0.04	0.28±0.07	0.19±0.02	0.34±0.06

**Table 3:** Results of the statistical analyses for differences in morphological traits between cryptic species.

Trait	Overall		Ireland		Denmark	
	*F_2,63_*	*P*	*F_2,31_*	*P*	*F_2,11_*	*P*
Thoracic width between the tegula	4.446	0.016	10.794	<0.001	1.57	0.251
Collar breadth on top of thorax (A)	0.295	0.746	3.132	0.058	1.567	0.252
A relative to body size	0.111	0.895	0.573	0.57	0.614	0.559
Length of collar extension below left tegula (B)	76.059	<0.001	61.638	<0.001	18.587	<0.001
Length of collar extension below right tegula (C)	71.046	<0.001	41.036	<0.001	7.188	0.01
Average length of collar extension below tegula (D)	93.378	<0.001	65.579	<0.001	12.974	0.001
B relative to body size	64.93	<0.001	47.209	<0.001	16.503	<0.001
C relative to body size	59.195	<0.001	30.886	<0.001	5.981	0.017
D relative to body size	77.282	<0.001	46.915	<0.001	10.812	0.003
Breadth of collar extension below left tegula (E)	29.667	<0.001	19.579	<0.001	40.088	<0.001
Breadth of collar extension below right tegula (F)	30.492	<0.001	22.158	<0.001	4.892	0.03
Average breadth of collar extension below tegula (G)	37.293	<0.001	22.913	<0.001	11.082	0.002
E relative to body size	21.466	<0.001	12.051	<0.001	26.005	<0.001
F relative to body size	21.754	<0.001	12.77	<0.001	3.81	0.055
G relative to body size	26.177	<0.001	13.437	<0.001	8.327	0.006

### Absolute collar breadth on top of thorax

There were no significant differences in absolute collar breadth on top of the thorax among species (Tables 2, 3). This was true for the entire data set, and for independent analyses of the Irish and Danish specimens (Tables 2, 3). Results were the same when collar breadth was re-scaled relative to body size (Tables 2, 3).

### Length of collar extension below tegula

As with body size, despite considerable overlap among species, there were significant differences among species for the length of the collar extension below the tegula on the left (*F*_2,63_ = 76.059, *P*<0.001, explaining 70% of the variance among specimens) and right (*F*_2,63_ = 71.046, *P*<0.001, explaining 69.3% of the variance) sides of the thorax (Table 2), as well as the average extension across both sides (*F*_2,63_ = 93.378, *P*<0.001, explaining 74.8% of the variance). All pairwise differences were significant (all *P*<0.001), with the collar of *B. magnus* extending significantly further below the tegula than that of *B. cryptarum*, which itself had a longer absolute extension of the collar than *B. lucorum* (Table 2). Results were qualitatively similar for the Irish and Danish data sets when analysed on their own (statistics not shown, Table 2), although in the Danish data, only *B. lucorum* had a consistently significantly shorter collar extension than *B. magnus*. Results were qualitatively the same when length of the collar extension was re-scaled relative to body size (Tables 2, 3).

### Breadth of collar extension below tegula

Again, despite large overlap among species in this character, there were significant differences in the breadth of the collar extension below the tegula on the left (*F*_2,63_ = 29.667, *P*<0.001, explaining 48.5% of the variance among specimens) and right (*F*_2,63_ = 30.492, *P*<0.001, explaining 49.2% of the variance) sides of the thorax (Table 2), as well as the average breadth across both sides (*F*_2,63_ = 37.293, *P*<0.001, explaining 54.2% of the variance). All pairwise differences were significant (all *P*<0.04) with the collar of *B. magnus* being broader below the tegula than that of *B. cryptarum*, which itself was broader than *B. lucorum* (Table 2). Results were qualitatively similar for the Irish and Danish data sets when analysed on their own (statistics not shown, Table 2), although in the Danish data only *B. lucorum* had a consistently significantly narrower collar extension than *B. magnus*. Results were qualitatively similar when breadth of the collar extension was re-scaled relative to body size (Tables 2, 3).

**Figure 3:**
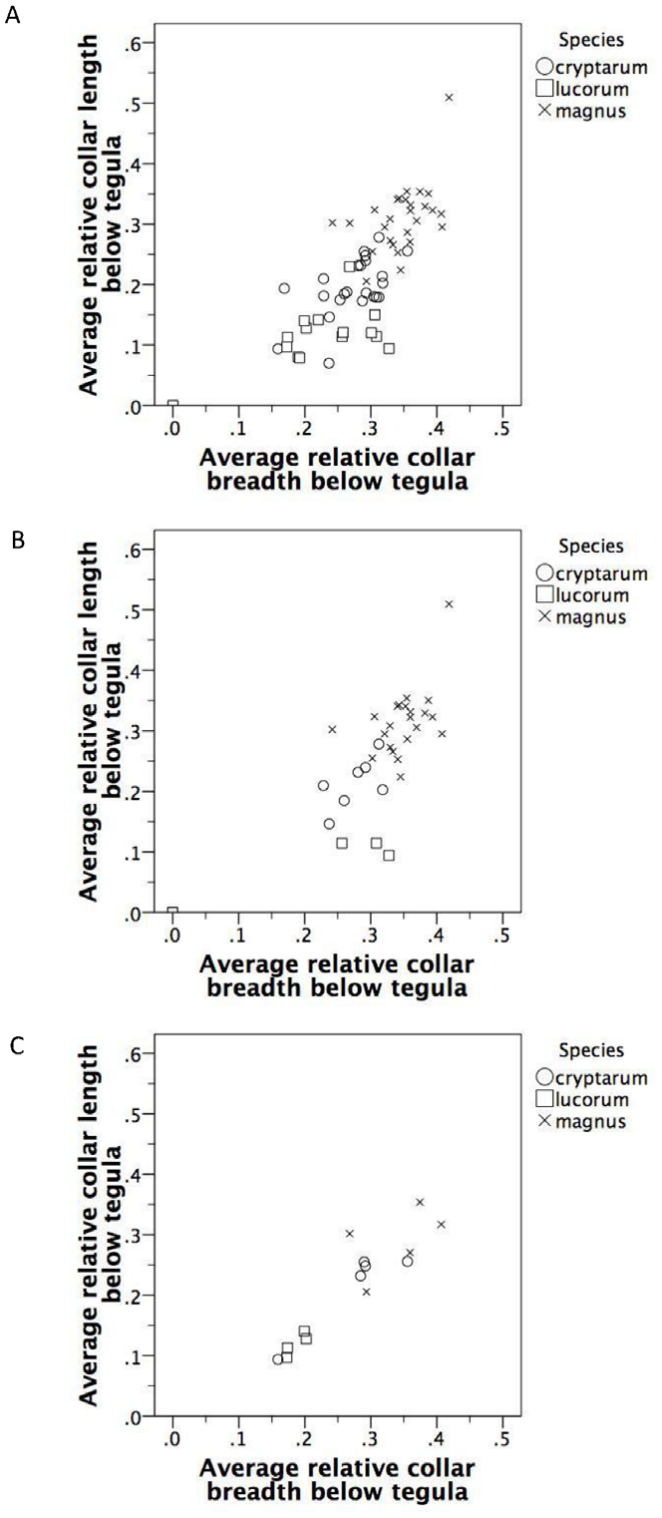
Combining the length and breadth of the collar extension of queens fails to distinguish between species (a) all samples combined, (b) for Irish specimens, and (c) for Danish specimens.

**Figure 4:**
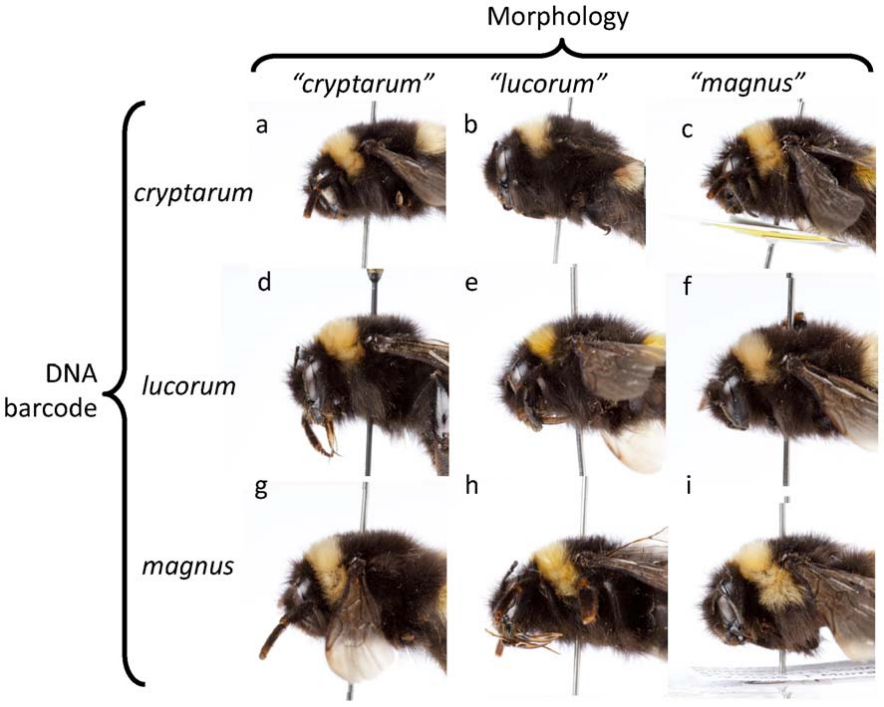
Morphological variation in queens of the three species as defined by COI barcode groups. Horizontal rows indicate species as identified by barcoding, whilst vertical columns show how molecularly identified specimens of each species can be morphologically identified as belonging to all 3 taxa. Specimens are identified by letters, which correspond to Table S1 as follows: a = T780, b = T773, c = T883, d = T875, e = T718, f = T787, g = T714, h = T713, i = T781.

One of the key characteristics that has been suggested as a way to identify the three species is the combination of relative breadth and length of the collar extension below the tegula. However, a plot of these two morphological variables against each other (Fig. 3) shows that they cannot unambiguously differentiate between the three species at either continental or local scales. At the European scale, all three species overlap in this combination of characters, with particularly strong overlap between *B. cryptarum*+*B. lucorum* and *B. cryptarum*+*B. magnus* respectively (Figure 3a). In the Danish samples, which were identified morphologically prior to molecular analyses, a similar degree of overlap is observed (Figure 3c). In contrast, the Irish samples suggest possible differentiation between *B. cryptarum* and *B. lucorum* using this combined character, although *B. cryptarum* and *B. magnus* still overlap (Figure 3b). Figure 4 shows examples of this overlap, with molecularly identified specimens from each species falling into all 3 of the colour-pattern groups.

### The “S-shape”

Queens of *B. cryptarum* were significantly more likely to have an “S-shape” than either *B. lucorum* or *B. magnus* (left-hand side of the thorax: *G* = 21.544, *P*<0.001; right-hand side of the thorax: *G* = 21.162, *P*<0.001). However, only 64% and 57% of *B. cryptarum* queens had the “S-shape” on the left and right sides, respectively (Table 4). While Irish queens were more likely to possess the S-shape if they were *B. cryptarum* (left: *G* = 7.488, *P* = 0.024; right: *G* = 6.678, *P* = 0.035), only 57% of *B. cryptarum* queens exhibited this marker while up to 18% of *B. magnus* possessed an S-shape, depending on thorax side (Table 4; see Figure 4, specimen g for an example of a *B. magnus* queen with an “S-shape”). Unsurprisingly, given that the animals were morphologically identified on the basis of the S-shape prior to sequencing, 100% of Danish *B. cryptarum* queens had the S-shape on the left and right sides of their thorax (*G* = 18.249, *P*<0.001 for both sides), while specimens of the other two species had no S-shape (Table 4).

**Table 4:** The presence/absence of morphological characters in molecularly identified queens of *Bombus cryptarum*, *B. lucorum* and *B. magnus*.

	All samples			Ireland samples			Denmark samples		
	*cryptarum*	*lucorum*	*magnus*	*cryptarum*	*lucorum*	*magnus*	*cryptarum*	*lucorum*	*magnus*
“S”-shape	14/22	1/16	4/27	4/7	0/5	4/22	5/5	0/4	0/5
Melanisation of collar	16/23	4/16	9/27	4/7	1/5	9/22	5/5	1/4	0/5
Extension beneath tegula	5/23	0/16	14/27	0/7	0/5	11/22	5/5	0/4	3/5

### Extension directly beneath the tegula

There was a significant difference among species in the possession of a collar that extended along the thorax below the tegula (left: *G* = 15.868, *P*<0.001; right: *G* = 25.490, *P*<0.001), a feature which is claimed to be diagnostic for *B. magnus*. While both *B. magnus* (48%) and *B. cryptarum* (22%) possessed this character state on the left side of the thorax, only *B. magnus* possessed it on the right side (44%) (Table 4). Only *B. magnus* queens from the Irish data set had a collar extension below the tegula. 45% of these queens had an extension on the left side, while 50% had it on the right side (*G* = 10.878, *P* = 0.004; *G* = 12.308, *P* = 0.002, respectively) (Table 4). There was significant variation among species in the Danish sample for the presence of a collar extension below the tegula on the left-hand side of the thorax (*G* = 12.391, *P* = 0.002). While 3 out of 5 *B. magnus* queens had this trait, all of the *B. cryptarum* queens possessed it (5/5). Only one of the Danish queens had a collar extension below the tegula on the right side, and this was a *B. magnus* (Table 4).

### Melanisation within the collar

There was a significant difference among species in the presence of melanised hairs scattered within the otherwise yellow collar (*G* = 9.890, *P* = 0.007; Table 4), another trait suggested to be diagnostic for *B. cryptarum*. Overall, 70% of *B. cryptarum* queens possessed this state, as opposed to 25% of *B. lucorum* queens and 33% of *B. magnus* queens. However, in the Irish dataset there was no relationship between melanisation and species (*G* = 1.738, *P* = 0.419; Table 4). Again, unsurprisingly, melanisation was present in all of the morphologically identified Danish *B. cryptarum* queens, only 1 of the *B. lucorum* queens and none of the *B. magnus* queens (*G* = 14.623, *P* = 0.001; Table 4).

## Discussion

Species determination is central to most assessments of biodiversity. Here we use DNA barcoding to show that currently recommended morphological traits for identifying a cryptic species complex of bumblebees are unreliable at both local and European scales. Below, we discuss these results in detail before assessing the impact they have on bumblebee conservation in western Europe.

COI barcode analysis indicated that three distinct molecular operational taxonomic units (MOTUs) exist within the European *B. lucorum* complex, a finding congruent with previous studies based on molecular genetics [20], [21], [22], [23] and secretions of the male labial glands [20]. Clade structure and genetic distances obtained in this study were similar to those obtained in previous studies [21] with intraspecific and interspecific genetic distance values of 0.001–0.004 and 0.033 to 0.044 respectively. Intraspecific and interspecific values obtained by Murray (2008) and Bertsch (2009) were 0.002–0.004 and 0.046–0.067 respectively. These distance values clearly indicate a significantly greater level of divergence between the three taxa and although the use of DNA sequences for species discovery or recognition is a contentious issue [57], [58], [59] our results are consistent with the view that the Northern European *B. lucorum* complex is comprised of three species.

The highest amount of intraspecific COI sequence variability was observed for *B. cryptarum,* a result consistent with previous studies [22]. There is a clear distinction between Finnish *B. cryptarum* and those sampled from Denmark, Scotland and Ireland. This differentiation, which corresponds to two distinct haplotypes, is supported by nine polymorphisms. This level of variation warrants further attention to evaluate the systematic significance of such divergence. A similar distinction was observed between Danish and Irish *B. magnus* with both forming monophyletic groups sister to each other. Danish *B. magnus* possessed two diagnostic polymorphisms with respect to their Irish counterparts (Table 1). *B. lucorum* demonstrated the lowest levels of intraspecific variation with only a single polymorphism observed across the Danish, Finnish, Irish and Orkney Isles specimens. These results may imply species differences in natural migration rates, and thus population structure across Europe, but this needs to be investigated using more appropriate markers (e.g., microsatellites). Such differences have obvious conservation implications given the level of habitat fragmentation in the European landscape.

The discrete differences between the three species obtained from the DNA barcode analysis were not replicated when morphological characters were analysed. Based on the characters most commonly cited as distinguishing these taxa, results from the morphological analyses failed to reveal a character that could reliably differentiate queens of the three species. Neither of the two most definitive character states – the presence of an S-shape on the collar, or extension of the collar under the wings – was consistently found in the species with which it has been associated as claimed previously [20]. While there were clearly significant differences among species in the presence of such traits, or in the mean values of more quantitative traits, there was significant overlap among species. While previous studies and bumblebee identification guides have claimed that specific morphological features can be used to identify queens of these three cryptic species, few [23], [28] have quantitatively examined such claims, and these studies had no independent diagnostic of species identification. Our results agree with Williams' [28] suggestion that these supposedly “diagnostic” characters overlap considerably and vary along a morphological continuum. Consequently, they are of limited use for morphological diagnosis of queens of *B. cryptarum*, *B. lucorum* and *B. magnus*.

The majority of characters used for bumblebee species differentiation in ecological studies are based on patterns relating to hair, which for bumblebees can be highly variable within species. Moreover, hair colour patterns often demonstrate a high degree of convergence and even Muellerian mimicry between species [15]. This can be clearly seen in our results (Fig. 4), where hair patterns are clearly variable and form a continuum across species.

In contrast to our study, previous molecular studies [20], [21] report specimens identified to species using COI sequencing that consistently display “species diagnostic” morphological characters. However these studies (and others) may have suffered from insufficient or selective sampling (it may be common for collectors to select ‘typical’ specimens for study, either intentionally or otherwise). The number of specimens included in the Bertsch et al's., study [20] was very low (*n* = 2 sequences per species). In total, 28 *lucorum* complex queens were included in the molecular analysis of Bertsch [21] and although 18 distinct geographic regions were represented in that study, the low sample size (max n = 4, with the majority of locations represented by n = 1) could not result in a conclusive evaluation of the morphological variance that exists within a given locality. The presence of clear geographical variation in colour patterns across species, as found in our study, suggests that additional investigations across the range of these species involving a randomly collected and large numbers of queens would be highly valuable.

The ability to conduct meaningful ecological and population genetic studies of particular taxa is reliant on our ability to correctly identify species. The results of our study clearly indicate that the potential for misidentification within the *B. lucorum* complex is high, particularly when dealing with randomly sampled large collections. Consequently, studies involving *B. lucorum s.l.* conducted prior to the utilisation of molecular methods for species identification may be subject to erroneous interpretation. The work of Waters et al [23] highlights the problems associated with field-based identifications of workers from the *B. lucorum* complex. The authors recommend the accompaniment of ecological studies with molecular methods for species identification. While this provides a solution, it is unlikely to be one that can be used for large-scale abundance and diversity studies. We hope that by demonstrating the problems with current characteristics used for queen species identification in the *B. lucorum* species complex, we will motivate the discovery of accurate morphological markers. Finally, we have shown that DNA barcoding for species identification can provide the basis for such quantitative studies.

## Supporting Information

Table S1 Accession information and geographic location of *B. lucorum* complex specimens utilised in the molecular and morphological analyses. Additional information for each specimen has been linked to the BOLD ID and deposited in the BOLD database. Abbreviations used: NBG: National Botanic Gardens (Dublin); NHM: Natural History Museum (London); TCD: Trinity College Dublin; UT: University of Turku (Finland).
(DOCX)
