# Supplementary material for: Colour Patterns Do Not Diagnose Species: Quantitative Evaluation of a DNA Barcoded Cryptic Bumblebee Complex
Source: PLoS One. 2012 Jan 6;7(1):e29251. doi: 10.1371/journal.pone.0029251 (PMC3253071; doi:10.1371/journal.pone.0029251)
Supplement: Table S1 — Accession information and geographic location of B. lucorum complex specimens utilised in the molecular and morphological analyses. Additional information for each specimen has been linked to the BOLD ID and deposited in the BOLD database. Abbreviations used: NBG: National Botanic Gardens (Dublin); NHM: Natural History Museum (London); TCD: Trinity College Dublin; UT: University of Turku (Finland). (DOCX) [file pone.0029251.s001.docx]

**Table S1** Accession information and geographic location of *B. lucorum* complex specimens utilised in the molecular and morphological analyses. Additional information for each specimen has been linked to the BOLD ID and deposited in the BOLD database. Abbreviations used: NBG: National Botanic Gardens (Dublin); NHM: Natural History Museum (London); TCD: Trinity College Dublin; UT: University of Turku (Finland).

| **Taxon^a^** | **Project ID** | **BOLD ID** | **GenBank Acc. No.** | **Locality** | **Reference Collection** |
| --- | --- | --- | --- | --- | --- |
| *B. cryptarum* | T698 | JCLUC001-11 | JN872587 | Powerscourt, Ireland | TCD |
|  | T707 | JCLUC002-11 | JN872586 | Clara, Ireland | TCD |
|  | T708 | JCLUC003-11 | JN872585 | Clara, Ireland | TCD |
|  | T709 | JCLUC004-11 | JN872584 | Powerscourt, Ireland | TCD |
|  | T723 | JCLUC005-11 | JN872583 | Gram Storskov, Denmark | NHM |
|  | T724 | JCLUC006-11 | JN872582 | Holstebro, Denmark | NHM |
|  | T725 | JCLUC007-11 | JN872581 | Isbjerg, Denmark | NHM |
|  | T726 | JCLUC008-11 | JN872580 | Stubbergaard Sø, Denmark | NHM |
|  | T727 | JCLUC009-11 | JN872579 | Stubbergaard Sø, Denmark | NHM |
|  | T766 | JCLUC010-11 | JN872578 | Powerscourt, Ireland | TCD |
|  | T768 | JCLUC011-11 | JN872577 | NBG, Ireland | TCD |
|  | T772 | JCLUC012-11 | JN872576 | Queenamidda, Orkney | NHM |
|  | T773 | JCLUC013-11 | JN872575 | Queenamidda, Orkney | NHM |
|  | T780 | JCLUC014-11 | JN872574 | Kippure, Ireland | TCD |
|  | T790 | JCLUC015-11 | JN872573 | Burray, Orkney Islands | NHM |
|  | T793 | JCLUC016-11 | JN872572 | Firth, Orkney Islands | NHM |
|  | T794 | JCLUC017-11 | JN872571 | Hardhill, Orkney Islands | NHM |
|  | T795 | JCLUC018-11 | JN872570 | St. Ola, Orkney Islands | NHM |
|  | T796 | JCLUC019-11 | JN872569 | Eday, Orkney Islands | NHM |
|  | T873 | JCLUC020-11 | JN872568 | Littoinen, Finland | UT |
|  | T876 | JCLUC021-11 | JN872567 | Houtskar, Finland | UT |
|  | T883 | JCLUC022-11 | JN872566 | Parainen, Finland | UT |
| *B. magnus* | T702 | JCLUC023-11 | JN872632 | Kippure, Ireland | TCD |
|  | T706 | JCLUC024-11 | JN872631 | Clara, Ireland | TCD |
|  | T710 | JCLUC025-11 | JN872630 | Kippure, Ireland | TCD |
|  | T712 | JCLUC026-11 | JN872629 | Powerscourt, Ireland | TCD |
|  | T713 | JCLUC027-11 | JN872628 | Kippure, Ireland | TCD |
|  | T714 | JCLUC028-11 | JN872627 | Killarney, Ireland | TCD |
|  | T715 | JCLUC029-11 | JN872626 | Killarney, Ireland | TCD |
|  | T717 | JCLUC030-11 | JN872625 | Killarney, Ireland | TCD |
|  | T733 | JCLUC031-11 | JN872624 | Randbøl Hede, Denmark | NHM |
|  | T734 | JCLUC032-11 | JN872623 | Vangså, Denmark | NHM |
|  | T735 | JCLUC033-11 | JN872622 | Holstebro, Denmark | NHM |
|  | T736 | JCLUC034-11 | JN872621 | Isbjerg, Denmark | NHM |
|  | T737 | JCLUC035-11 | JN872620 | Randbøl Hede, Denmark | NHM |
|  | T762 | JCLUC036-11 | JN872619 | Powerscourt, Ireland | TCD |
|  | T763 | JCLUC037-11 | JN872618 | Powerscourt, Ireland | TCD |
|  | T764 | JCLUC038-11 | JN872617 | Bahana Woods, Ireland | TCD |
|  | T765 | JCLUC039-11 | JN872616 | Killarney, Ireland | TCD |
|  | T769 | JCLUC040-11 | JN872615 | Killarney, Ireland | TCD |
|  | T770 | JCLUC041-11 | JN872614 | Powerscourt, Ireland | TCD |
|  | T771 | JCLUC042-11 | JN872613 | Killarney, Ireland | TCD |
|  | T781 | JCLUC043-11 | JN872612 | Killarney, Ireland | TCD |
|  | T782 | JCLUC044-11 | JN872611 | Killarney, Ireland | TCD |
|  | T783 | JCLUC045-11 | JN872610 | Powerscourt, Ireland | TCD |
|  | T784 | JCLUC046-11 | JN872609 | Powerscourt, Ireland | TCD |
|  | T785 | JCLUC047-11 | JN872608 | Killarney, Ireland | TCD |
|  | T788 | JCLUC048-11 | JN872607 | Kippure, Ireland | TCD |
|  | T789 | JCLUC049-11 | JN872606 | Powerscourt, Ireland | TCD |
| *B. lucorum* | T690 | JCLUC050-11 | JN872605 | Powerscourt, Ireland | TCD |
|  | T691 | JCLUC051-11 | JN872604 | Clara, Ireland | TCD |
|  | T692 | JCLUC052-11 | JN872603 | Killarney, Ireland | TCD |
|  | T716 | JCLUC053-11 | JN872602 | Powerscourt, Ireland | TCD |
|  | T718 | JCLUC054-11 | JN872601 | Powerscourt, Ireland | TCD |
|  | T728 | JCLUC055-11 | JN872600 | Stubbergaard Sø, Denmark | NHM |
|  | T729 | JCLUC056-11 | JN872599 | Holstebro, Denmark | NHM |
|  | T730 | JCLUC057-11 | JN872598 | Eshøj Plantage, Denmark | NHM |
|  | T731 | JCLUC058-11 | JN872597 | Gram Storskov, Denmark | NHM |
|  | T767 | JCLUC059-11 | JN872596 | NBG, Ireland | TCD |
|  | T787 | JCLUC060-11 | JN872595 | Killarney, Ireland | TCD |
|  | T791 | JCLUC061-11 | JN872594 | Netherborough, Orkney Islands | NHM |
|  | T792 | JCLUC062-11 | JN872593 | Burray, Orkney Islands | NHM |
|  | T875 | JCLUC063-11 | JN872592 | Houtskar, Finland | UT |
|  | T880 | JCLUC064-11 | JN872591 | Turku, Finland | UT |
|  | T887 | JCLUC065-11 | JN872590 | Pesa, Finland | UT |
|  | T904 | JCLUC066-11 | JN872589 | Naantali, Finland | UT |
|  | T909 | JCLUC067-11 | JN872588 | Kultaranta, Finland | UT |

^a^ Species identification based on COI sequence analysis
